# Automated Video Behavior Recognition of Pigs Using Two-Stream Convolutional Networks

**DOI:** 10.3390/s20041085

**Published:** 2020-02-17

**Authors:** Kaifeng Zhang, Dan Li, Jiayun Huang, Yifei Chen

**Affiliations:** College of Information and Electrical Engineering, China Agricultural University, Beijing 100083, China; s20183081343@cau.edu.cn (K.Z.); bs20173080602@cau.edu.cn (D.L.); s20183081301@cau.edu.cn (J.H.)

**Keywords:** pig behavior, two-stream convolutional network, deep learning, inflated 3D convnet, temporal segment networks

## Abstract

The detection of pig behavior helps detect abnormal conditions such as diseases and dangerous movements in a timely and effective manner, which plays an important role in ensuring the health and well-being of pigs. Monitoring pig behavior by staff is time consuming, subjective, and impractical. Therefore, there is an urgent need to implement methods for identifying pig behavior automatically. In recent years, deep learning has been gradually applied to the study of pig behavior recognition. Existing studies judge the behavior of the pig only based on the posture of the pig in a still image frame, without considering the motion information of the behavior. However, optical flow can well reflect the motion information. Thus, this study took image frames and optical flow from videos as two-stream input objects to fully extract the temporal and spatial behavioral characteristics. Two-stream convolutional network models based on deep learning were proposed, including inflated 3D convnet (I3D) and temporal segment networks (TSN) whose feature extraction network is Residual Network (ResNet) or the Inception architecture (e.g., Inception with Batch Normalization (BN-Inception), InceptionV3, InceptionV4, or InceptionResNetV2) to achieve pig behavior recognition. A standard pig video behavior dataset that included 1000 videos of feeding, lying, walking, scratching and mounting from five kinds of different behavioral actions of pigs under natural conditions was created. The dataset was used to train and test the proposed models, and a series of comparative experiments were conducted. The experimental results showed that the TSN model whose feature extraction network was ResNet101 was able to recognize pig feeding, lying, walking, scratching, and mounting behaviors with a higher average of 98.99%, and the average recognition time of each video was 0.3163 s. The TSN model (ResNet101) is superior to the other models in solving the task of pig behavior recognition.

## 1. Introduction

Pig behavior reflects the animal’s welfare status, well-being conditions, and social interactions [1,2]. Appropriate feeding behavior can ensure the healthy growth of pigs and help determine their food intake. Reduction in food intake means that pig health and welfare are compromised [3,4] and can be considered as a signal for alarming suspected cases [1]. Walking and lying behaviors can reflect the activity level of pigs. During illness, pigs generally reduce activity, posture in protective positions, and increase lying duration [5,6]. Estrus can be estimated through pig mounting behavior [7], which can cause bruises, lameness, stress, and leg fractures [8,9]. So, the timely detection and intervention of mounting behavior can increase animal welfare and further ensure pig health [10]. Pig scratching behavior is the grooming behavior whose function is mainly to reduce or eliminate external stimuli such as parasites, flies, mosquitoes, and dirt. The infection of skin diseases can be evaluated by observing the pruritus index of pigs [11,12]. Pig health and welfare compromises can be detected early through detecting pig behavior [13]. Therefore, monitoring the behavior of pigs and timely intervention that could help to keep the pigs in normal conditions is especially important.

Monitoring pig behavior by staff is time consuming, subjective, and impractical [2,14]. The method of monitoring pig behavior data through sensors also has certain disadvantages. Most sensors are attached to the surface of pigs, which can easily cause pigs’ stress response [15] and alter the normal behavior of pigs [2]. Staff also need to record sensor readings frequently, which is more troublesome. So, contactless, low-cost, easy, and effective computer vision techniques [1] have been widely used in animal monitoring processes and play an essential role in assessment of animal behavior [16]. Viazzi et al. [17] extracted the mean intensity of motion and the occupation index; then, they used the Linear Discriminant Analysis (LDA) to classify two features to identify aggressive behavior with an accuracy of 89%. Kashiha et al. [18] made the pig into an ellipse. Pig locomotion behavior was calculated through ellipse displacement. Nasirahmadi et al. [8] also made the pig into an ellipse. If the long axis of the ellipse was 1.3 to 2 times of the length of the normal ellipse long axis and the short axis was 1.3 to 1.8 times of the length of the normal ellipse short axis, it was determined that mounting behavior occurred. The accuracy of the method was 92.7%. Some studies in the literature [19,20] detected behavior through the distance between a part of the pig body and the object such as drinking nipple and feeder. Image contour analysis [19] was used to detect distances between pig head, ears, and drinking nipple and the duration that a pig stayed at the drinking nipple was calculated, which helped judge drinking behavior with an accuracy of 92%. Lao et al. [20] obtained the necessary feature values for identification of the sow’s behaviors by depth image data. When the head of a pig was in the feeder with up and down movement, feeding behavior could be determined. This method had a 97.4% accuracy rate for feeding and 92.7% accuracy rate for drinking. The above methods of behavioral feature extraction rely on manual observation and design and high-precision image segmentation, so these methods have higher requirements on the pigpen environment and shooting conditions. Deep learning can solve these problems.

Deep learning, which is an excellent research method in computer vision techniques, would be widely applied in the study of the animal behavior community [21]. Some methods detect pigs from images through target detection network models to perform pig behavior recognition. Zheng et al. [2] and Yang et al. [22] used Faster Region-Convolutional Neural Networks (Faster-RCNN), which can detect pigs effectively to recognize pig postures and feeding behaviors. Sows were segmented from all frames through the Fully Convolutional Network (FCN) model, which could help recognize sows’ nursing behavior with an accuracy of 97.6% [23]. Nasirahmadi et al. [16] proposed three detector methods including Faster R-CNN, single shot multibox detector (SSD), and region-based fully convolutional network (R-FCN) to recognize the standing, lying on side, and lying on belly postures of pigs with a mean average precision (mAP) of over 0.93. Real-time sow drinking, urination, and mounting behavior recognition has been achieved by using an optimized target detection method based on the SSD and the MobileNet [24]. Real-time recognition speed could reach 7 frames per second, and the mAP was 93.4%. Mask Region-Convolutional Neural Networks (Mask R-CNN) was also used as a pig segmentation network [10]. Then, the eigenvectors, which included the region of interest (RoI) parameters and mask coordinates, could be extracted. Kernel extreme learning machine (KELM) was applied to a classifier for eigenvectors to output the results regarding whether mounting behavior had occurred. The method was able to identify mounting behavior effectively with an accuracy of 91.47%.

Existing deep learning methods recognize pig behavior based on still image frames that only contain spatial information, which cannot effectively obtain the coherent temporal information of the behavior. The temporal information of the behaviors between consecutive frames is also important for behavior recognition. The two-stream convolutional network [25] is a classical framework in the field of deep learning behavior recognition, and it is composed of a spatial stream network and a temporal stream network that can extract the spatial and temporal information of the videos, respectively. Therefore, in this paper, a standard pig video behavior dataset was created and two-stream convolutional network models, including inflated 3D convnet (I3D) [26] and temporal segment networks (TSN) [27], were proposed to extract the spatial and temporal information from videos instead of still images to achieve pig five kinds of different behavior recognition.

## 2. Materials and Methods

### 2.1. Establishment of Pig Video Dataset

#### 2.1.1. Data Acquisition

**Figure 1:**
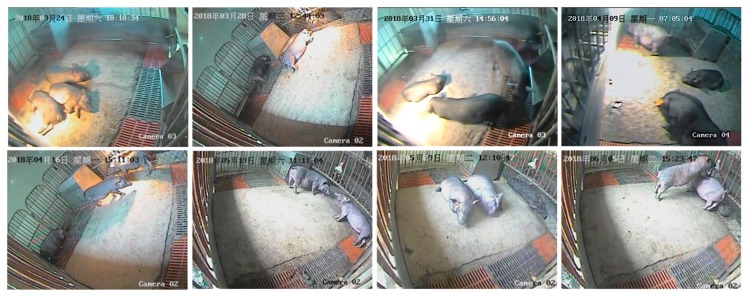
Sample frames from a video sequence that contains the daily behaviors of pigs.

The experiment was conducted at the Zhuozhou Breeding Base of China Agricultural University. It took 80 days from 23 March 2018 to 13 June 2018 to collect videos of different daily behaviors of pigs. The size of the pigpen was 2 m × 2 m. There were two or three fragrance pigs in each pigpen. Four pigpens were selected for collecting pig behavior videos. Each pigpen was equipped with a fixed Sony infrared camera SSC-CB565R for shooting videos. The height of the camera from the ground was 1 m, and four cameras shot videos of pigs in four pigpens at the same time. The videos were captured in a 1280 × 1024 pixel spatial resolution at 25 fps and transferred to the Hikvision Digital Video Recorder (DVR) DS-8104HF-ST. With the increase of time, the age and body size of pigs were also increasing. The lighting conditions of the pigpens were also different at different times. Different pigpen environments from four pigpens, pig ages, pig body sizes, and lighting conditions can well test the robustness of the proposed model. Sample frames from the video sequence are shown in Figure 1.

#### 2.1.2. Dataset Construction and Pre-Processing

**Figure 2:**
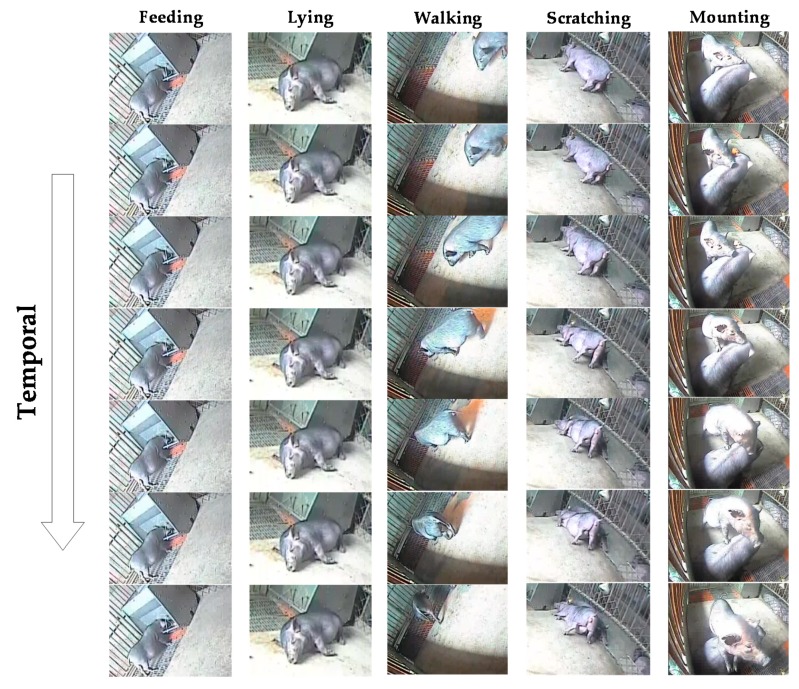
Sample image frames of 5 kinds of behaviors in the dataset.

All the collected videos were transferred to the computer through a mobile hard disk, and the daily behaviors of the pigs were observed. The five kinds of behaviors of feeding, lying, walking, scratching, and mounting were chosen to identify. The five kinds of behaviors occurred in the natural pigpen environment without intervention. The collected videos were filtered to remove the blurred videos caused by poor light and the videos containing invalid pictures. The software video editing king was used to cut and edit the videos, and representative video clips containing five kinds of behaviors of pigs were selected. In order to make the trained network more robust, we tried to select video clips from different shooting periods with different poses for the same behavior and avoided the unity of different samples of the same behavior. Each edited clip contained one behavior. The length of the clips was 6.276 s on average. Videos that are too long may be mixed with too much invalid information, which will affect the recognition results, while too short videos may miss key information in the entire set of behavior. The total duration of the clips was 104.6 min. The videos were exported to a sequence of video frames with 320 × 240 pixels and frame rate of 25 fps after editing. Videos were categorized by actions and uniformly named by “action name + number”. There were 200 videos for each behavior and a total of 1000 Audio Video Interleaved (AVI) videos, which had five pig behavior categories. The specific video parameters of the dataset and the scale of the dataset are shown in Table 1. Sample image frames of five kinds of behaviors in the dataset are shown in Figure 2. The video samples of the dataset included scale changes and lighting changes, and the background was diverse. The poses of the pigs in the video samples of the same behavior were not consistent, which reflected the real situation in the pigpen scene.

**Figure 3:**
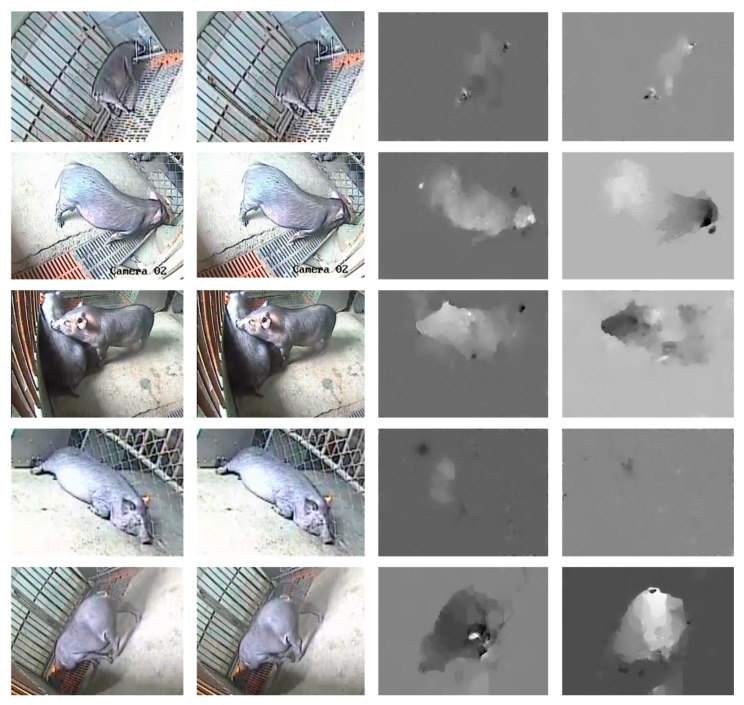
Two consecutive video frames and corresponding optical flow diagram (x, y direction).

The motion information in the videos is very important for behavior recognition, and the optical flow diagram can well reflect the motion information such as the direction and speed of the moving target. The RGB color mode (RGB) images in the dataset were used as the input of the spatial convolution network to extract the appearance information, and the corresponding optical flow diagram was used as the input of the temporal convolution network to extract the motion information. The results of the two streams were fused to obtain the video behavior classification results. The TVL1 optical flow algorithm [28] implemented by OpenCV was used to obtain the optical flow values in the horizontal × direction and the vertical y direction. Then, the optical flow fields were discretized into the interval from 0 to 255 by a linear transformation. Two consecutive video frames and the corresponding optical flow diagram (x, y direction) are shown in Figure 3.

### 2.2. Two-Stream Convolutional Network Models

In this study, two two-stream convolutional network models were developed for pig multi-behavior recognition, including temporal segment networks model and an inflated 3D convnet model. For the temporal segment networks model, we chose the Inception architecture and the ResNet architecture as backbone networks to study and compare the performance of the models.

#### 2.2.1. Temporal Segment Networks Model

**Figure 4:**
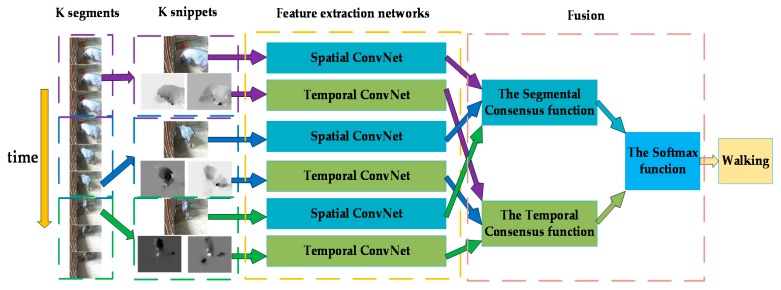
Temporal segment network (TSN) network structure.

Two-stream convolutional networks proposed by [25] randomly sample a single frame (the spatial network) or a single stack of frames (the temporal network) from the video to be input, which will lead to the inability to convey the information of the entire video effectively. The TSN model, which is also a two-stream convolutional network framework, was proposed by Wang et al. in 2016 to obtain more video context information. TSN has a sparse temporal sampling strategy and firstly divides the video that will be predicted into multiple non-overlapping segments of the same length. Then, a short snippet is randomly selected from each segment. Each snippet is input into the networks for feature extraction and generates its own preliminary prediction for the behavior category. The behavior categorical scores of several snippets will be fused by a segmental consensus function, which achieves segmental consensus. Finally, the prediction results of the two channels are mixed to obtain the final video-level prediction. The TSN network structure is shown in Figure 4.

As the figure above shows, the video V that will be classified is divided into K segments $\left\{S_{1},S_{2},\ldots ,S_{K}\right\}$ according to the number of video frames. $\left\{T_{1},T_{2},\ldots ,T_{K}\right\}$ is randomly sampled from corresponding segments; $\left\{S_{1},S_{2},\ldots ,S_{K}\right\}$ represents a set of K snippets. The TSN model that models $\left\{T_{1},T_{2},\ldots ,T_{K}\right\}$ is as follows:

(1) $$TSN\left(T_{1},T_{2},\ldots ,T_{K}\right)\;\;=\;H\left(G\left(F\left(T_{1};W\right),F\left(T_{2};W\right),\ldots ,F\left(T_{K};W\right)\right)\right)$$

$F\left(T_{K};W\right)$ represents that the convolutional neural network with parameters W extracts the feature vectors of $T_{K}$ and generates categorical scores. The spatial convolutional network shares a set of network parameters W over K snippets, as does the temporal convolutional network. G is the segmental consensus function, which fuses the outputs of the K networks in a certain way and outputs the consensus of the category scores. The Softmax function that is adopted as the function H obtains the probability of each type of behavior that the input video sample is classified into.

(2) $$H\left(G_{i}\right)\;=\;\frac{e^{G_{i}}}{\sum_{a=1}^{C}e^{G_{a}}},\;i\;=\;1,2,\ldots ,C$$

According to standard categorical cross-entropy loss function, the final loss function for the segmental consensus $G\;=\;G\left(F\left(T_{1};W\right),F\left(T_{2};W\right),\ldots ,F\left(T_{K};W\right)\right)$ is defined as

(3) $$L\left(y,G\right)\;=\;-\overset{C}{\underset{i=1}{\sum }}y_{i}(G_{i}-\log\overset{C}{\underset{j=1}{\sum }}e^{G_{j}})$$

(4) $$G_{i}\;=\;g\left(F_{i}\left(T_{1}\right),F_{i}\left(T_{2}\right),\ldots ,F_{i}\left(T_{K}\right)\right),\;i\;=\;1,2,\ldots ,C$$

where C is the number of behavior categories, $y_{i}$ is the groundtruth label of behavior category i, and the aggregation function g is evenly averaging. During the back-propagation, the gradient of W with respect to L is calculated as

(5) $$\frac{\partial L\left(y,G\right)}{\partial W}\;=\;\frac{\partial L}{\partial G}\sum_{k=1}^{K}\frac{\partial G}{\partial F\left(T_{k}\right)}\frac{\partial F\left(T_{k}\right)}{\partial W}.$$

We use the stochastic gradient descent (SGD) optimization method. The video-level prediction loss value L is back-propagated to update the model parameters W after multiple iterations.

Based on the temporal segment network framework mentioned above, we compared the performance of several deep learning network models as backbone networks (e.g., BN-Inception [29], ResNet [30], InceptionV3 [31], InceptionV4 [32], or InceptionResNetV2 [32]). Szegedy et al. [33] proposed a convolutional network structure for inception. The inception model runs filters with multiple sizes (e.g., 1 × 1, 3 × 3 or 5 × 5) on the same level and performs multiple convolution or pooling operations on the input images in parallel, which causes the network to become slightly wider, not deeper. Processing these operations in parallel and combining all the results will get different scale features of the images. For BN-Inception, BN is Batch Normalization, which addresses the problem of internal covariate shift [29]. Applying batch normalization to the inception model, it can reduce the training steps’ times, reduce the use of dropout, and obtain a good balance between accuracy and efficiency [27]. In addition, BN-Inception replaces the 5 × 5 convolution layers in the inception module with two 3 × 3 convolution layers [29], reducing the amount of parameters and speeding up the calculation speed. InceptionV3 is proposed to improve the inception structure for better performance. It factorizes the convolution kernel size, uses auxiliary classifiers, and adopts efficient grid size reduction [31]. InceptionV4 is an extension of InceptionV3, which is deeper and wider and has more Inception modules than InceptionV3 [32]. The InceptionResNetV2 model is a combination of the Inception architecture and residual connections, which can significantly accelerate the training of Inception networks [32].

Three architectures of the ResNet model (e.g., ResNet18, ResNet50, and ResNet101) were also implemented as feature extractors of the pig video behavior frames. ResNet has a deep residual learning framework that was introduced by [30] to solve the degradation problem that occurs when deeper networks are able to start converging. The core idea of ResNet is to introduce identity shortcut connections that skip one or more layers directly. The entire network only needs to learn the difference between input and output, simplifying the learning difficulty. There are two types of residual modules in ResNet. One is to concatenate two 3 × 3 convolution layers together as a residual module. The other is that three convolutional layers of 1 × 1, 3 × 3, and 1 × 1 are concatenated together as a residual module. ResNet18, ResNet50, and ResNet101 are all formed by stacking the residual modules. The specific network structure of ResNet is shown in Table 2.

#### 2.2.2. Inflated 3D ConvNet Model

**Figure 5:**
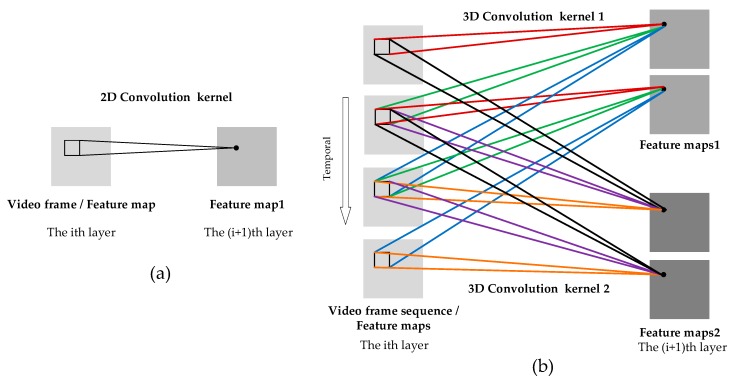
The comparison Figure of 2D convolution and 3D convolution. (**a**) 2D convolution diagram: the 2D convolution kernel convolves with a single image to obtain a 2D feature map. (**b**) 3D convolution diagram: the input is a 3D cube composed of multiple consecutive video frames that can be expanded into multiple 2D images in temporal series. The size of the 3D convolution kernel in the temporal dimension is 3. The 3D convolution kernel convolves with multiple consecutive video frames to obtain multiple feature maps. The connecting lines of shared weights are in the same color. Two different 3D convolution kernels can extract two types of features and generate two sets of different feature maps on the right.

The I3D network is a new type of two-stream 3D convolutional neural network proposed by [26]. The 3D convolutional neural network was originally proposed by [34]. The process of 2D convolution is to convolve the image and the 2D convolution kernel to extract the spatial features of the image, while the process of 3D convolution is to convolve the cube formed by stacking multiple consecutive video frames and the 3D convolution kernel to extract video features in spatio-temporal dimension. The comparison figure of the two is shown in Figure 5. For 3D convolution, the value of a certain location in a feature map is obtained by convolving with the local receptive fields at the same position in three consecutive frames of the previous layer. The feature maps are connected to multiple consecutive frames of the previous layer, so they can capture video motion information. Each 3D convolution kernel can extract one type of feature. If we choose different 3D convolution kernels to convolve with three consecutive frames, we can extract multiple spatial and temporal features of the video. The original input are continuous video frames, and feature maps are generated after 3D convolution; then, these feature maps in the previous layer are convolved to generate new feature maps in the next layer. The value of the unit with coordinates (x, y, z) in the feature map is given by the following formula:

(6) $$v_{ij}^{xyz}\;=\;f(b_{ij}+\underset{m}{\sum }\overset{P_{i}-1}{\underset{p=0}{\sum }}\overset{Q_{i}-1}{\underset{q=0}{\sum }}\overset{R_{i}-1}{\underset{r=0}{\sum }}w_{ijm}^{pqr}v_{\left(i-1\right)m}^{\left(x+p\right)\left(y+q\right)\left(z+r\right)})$$

where $w_{ijm}^{pqr}$ represents the (p,q,r)th weight of the 3D convolution kernel connected with the mth feature map in the previous layer, $b_{ij}$ is the offset, and the 3D convolution kernel size is $P_{i}\;\times \;Q_{i}\;\times \;R_{i}$. Function f is the activation function.

**Figure 6:**
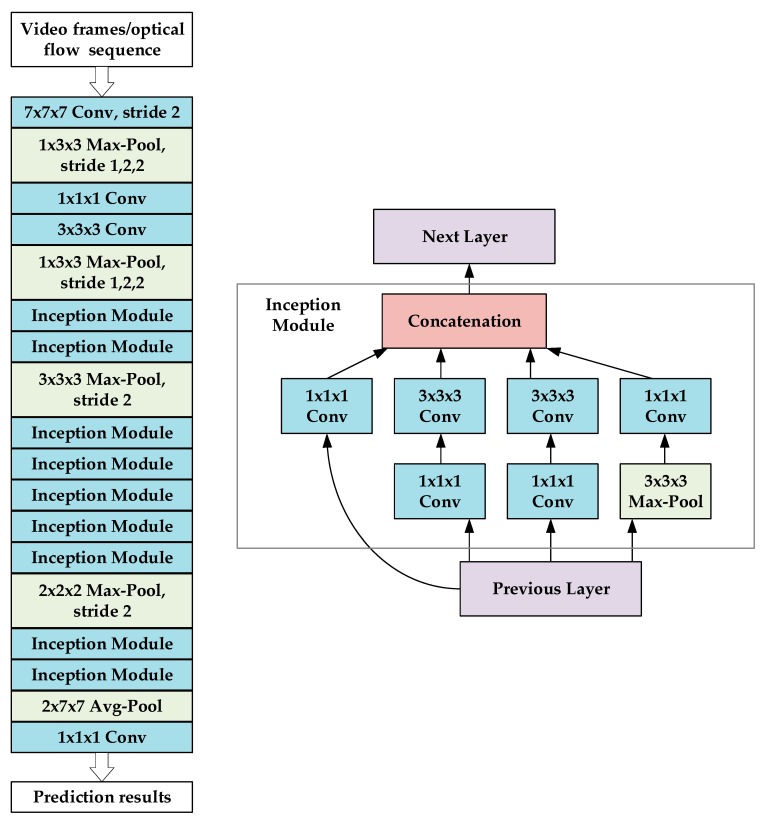
The overall structure of Inflated 3D ConvNet.

The I3D model is inflated from a 2D convolutional neural network InceptionV1 [33]. For the InceptionV1 model, all N × N 2D filters and pooling kernels add a time dimension into N × N × N 3D convolution kernels. Network parameter initialization can be performed by using pre-trained parameters on ImageNet [35], which repeats the weights of the 2D convolution kernels N times along the time dimension and normalizes them by dividing by N. It may not be appropriate to inflate all N × N 2D kernels to N × N × N 3D kernels, and the influence of factors such as frame rate and image size must also be considered. If the size of the time dimension is too large, the edge characteristics of the object may be destroyed. If the size is too small, dynamic information may not be captured well. Therefore, the kernels of the initial two max-pooling layers of the network are 1 × 3 × 3, and the strides are 1 in the time dimension to maintain the features extracted by the shallow network. The kernel of the final average-pooling layer is 2 × 7 × 7 with the stride of 2 in the time dimension. The overall structure of I3D and the Inflated InceptionV1 (Inception Module) structure are shown in Figure 6.

In addition, a Rectified Linear Unit (ReLU) function that is used as a non-linear activation function and a Batch Normalization (BN) [29] layer that is used to renormalize the activation values of the previous layer to accelerate network convergence are followed after each convolution layer except for the last convolutional layer.

**Figure 7:**
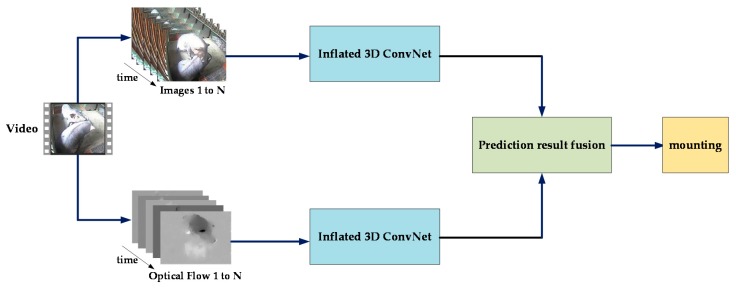
The network architecture of two-stream inflated 3D ConvNets.

RGB video frames and stacked optical flow are input into two I3D convolutional neural networks to extract the temporal and spatial information of the video respectively, and then the classification results of the two networks are fused to get final results. The network architecture of Two-Stream Inflated 3D ConvNets is shown in Figure 7.

## 3. Experiment and Discussion

### 3.1. Experiment Implementation Details

#### 3.1.1. Experimental Environment

The model comparison experiments conducted in this article were all performed on the server DELL R730. The processor was Xeon E5-2667, the main frequency was 3.2 GHz, and the memory was 128 GB. The hard disk had one 1 T solid-state disk and two 2 T mechanical hard disks, and the Graphic Processing Unit (GPU) was GTX 1080 of 8G video memory. The core software resources included an Ubuntu 16.04 operating system, version 9.0.176 of CUDA, and version 7.0.5 of cuDNN. TensorFlow1.10.0 and pytorch 1.2.0 deep learning framework were also used to accomplish these experiments. OpenCV was used for video processing.

#### 3.1.2. Experimental Parameter Setting

For the TSN model, the spatial network weights were initialized with pre-trained models from ImageNet [35]. For the temporal network, cross modality pre-training, which copies the average of the weights on the RGB channels according to the number of input channels of the temporal network, was adopted. The optimization method was a stochastic gradient descent algorithm. The batch size was set as 8, the momentum was 0.9, and the initial learning rate was 0.001. For the spatial stream convnets, the dropout ratio was 0.8 and the epochs were 80. For the temporal stream convnets of the TSN model, the dropout ratio was 0.7 and the epochs were 340. The input size of the video frames and the optical flow diagrams was 224 × 224. During training, the number of snippets K was 3. We sampled 25 RGB frames or optical flow stacks from the action video for testing.

For the I3D model, the weights of the I3D network were initialized with pre-trained models from ImageNet and Kinetics. The optimization method was also a stochastic gradient descent algorithm. The batch size was set as 6, the momentum was 0.9, and the initial learning rate was 0.0001. During training and testing, input images were randomly cropped to 224 × 224, and we set the number of input frames, which was 16. Too many frames will cause excessive memory usage during training, and too few frames will cause insufficient information extraction. The maximum number of iterations was 10,000 for the spatial stream convnets, and the maximum number of iterations was 20,000 for the temporal stream convnets.

### 3.2. Results and Discussion of the Experiments

The dataset was divided into a training set and test set in a 4:1 ratio randomly in a non-overlapping manner. The training set contained 773 videos and the test set contained 227 videos. The stochastic selected 227 test samples were input into the I3D and TSN networks, which were trained by the training set, and then we obtained the accuracy of each category of the videos.

For the TSN model, if K is 1, the networks will degenerate into normal two-stream networks. Table 3 shows the behavior recognition accuracy of TSN models with different backbone networks under different numbers of video segments. It can be seen from Table 3 that the behavior recognition performance is better when the video is divided into three segments. The video information can be fully extracted, and actions can be modeled from the whole video. The experimental results reflect the superiority of the sparse temporal sampling strategy.

Table 4 shows the performance of different network architectures of two-stream convolutional networks. It can be seen from Table 4 that the behavior recognition performance is best when the backbone network is ResNet101 in both spatial and temporal networks, which shows that the recognition accuracy can be improved by increasing the depth of the network. Residual network solves the problem of network degradation caused by an overly deep network. It also reflects the superiority of the combination of two-stream networks and deep networks. At the same time, it can be concluded that the accuracy of the temporal networks is higher than the accuracy of the spatial networks. From this, we can see the importance of optical flow, which has motion characteristics and apparent invariance [36] for recognition. The introduction of optical flow has significantly improved the fused accuracy, which proves that the two-stream networks have certain complementarity. Table 5 shows the confusion matrix of the TSN model whose feature extraction network is ResNet101. It can be seen from the table that the recognition accuracy of feeding, mounting, and lying reaches 100%, while the recognition accuracies of scratching and walking are 97.82% and 97.14%, respectively. The average recognition accuracy is 98.99% and the model works well in pig behavior recognition.

**Figure 8:**
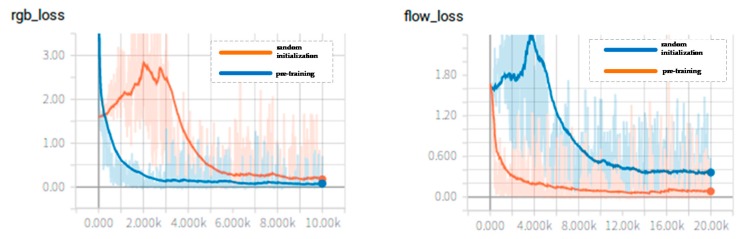
The change curve of the loss value of the two-stream convolutional networks: the picture on the left is for the spatial convnets, and the picture on the right is for the temporal convnets. The horizontal axis of the loss value curve figures represents the maximum number of iterations, and the vertical axis represents the loss value.

**Figure 9:**
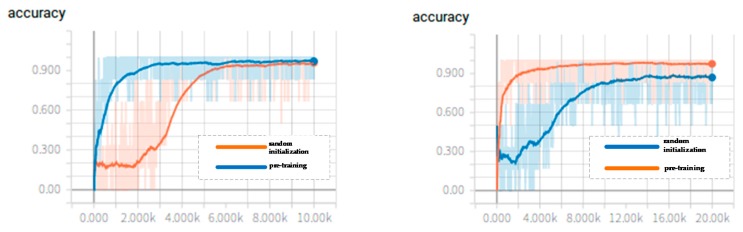
The change curve of the accuracy rate of the two-stream convolutional networks: the picture on the left is for the spatial convnets, and the picture on the right is for the temporal convnets. The horizontal axis of the accuracy rate curve figures represents the maximum number of iterations, and the vertical axis represents the accuracy.

For the I3D model, we compared two ways of initializing the feature extraction network parameters. One was to expand the 2D convolution kernel parameters from Imagenet to 3D convolution kernel parameters, and then we further used the I3D network parameters from the Kinetics dataset to fine-tune the network with the pig video behavior dataset. The other one was to set the parameters of the feature extraction network through completely random initialization and then directly train the network with the pig video behavior dataset. The change curves of the loss function values of two-stream convolutional networks are shown in Figure 8, and the change curve of the accuracy rate of two-stream convolutional networks during training are shown in Figure 9. For comparing the parameter initialization methods, the curves of the networks with two different parameter initialization methods are all drawn in the same graph.

It can be seen from Figure 8 that, whether the networks are the temporal convnets or the spatial convnets, the networks with randomly initialized parameters may have large fluctuations in the loss value during the initial training phase. However, as the number of training iterations increases, the model will eventually reach a convergence state. However, the convergence speed of the networks with randomly initialized parameters is slower than the convergence speed of the networks with pre-training. According to Figure 9 the networks with pre-training have higher recognition accuracy and faster convergence speed. Through the analysis above, the way that the pre-trained models are applied and then the parameters are fine-tuned according to the new pig dataset accelerates the convergence speed and achieves a high accuracy. The I3D networks were tested with the test dataset, and the result is shown in Table 6. It can also be concluded that the accuracy of the temporal networks is higher than the accuracy of the spatial networks. The model is more sensitive to optical flow information. Compared with the single stream network, the two-stream network still shows better performance. Table 7 shows the confusion matrix of the I3D model with pre-training. One scratching behavior was misidentified as walking behavior, and one walking behavior was misidentified as feeding. The average recognition accuracy is also 98.99%.

**Figure 10:**
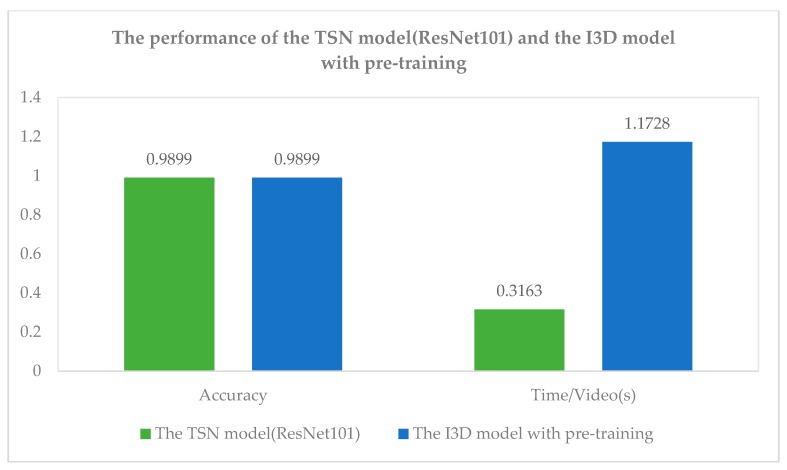
The performance of the TSN model (ResNet101) and the I3D model with pre-training.

Through the experiments above, we can find that the TSN model whose feature extraction network is ResNet101 and the I3D model with pre-training all have achieved high accuracy rate which is 98.99% in pig video behavior recognition. In order to compare two models more comprehensively, we compared the average recognition time of each video. The result is shown in Figure 10.

It can be known from the results that the average recognition time of each video in the TSN network (ResNet101) is 0.8565 s less than that of the I3D network with pre-training when the accuracy of the two models is the same. Therefore, the TSN model (ResNet101) has good recognition efficiency and recognition effect for multi-behavior recognition of pigs and has good robustness for different pigpen environments, pig ages, pig body sizes, and lighting conditions.

They are lots of publications in the pig behavior recognition field.

The literatures [17,18,19,20] are all pig behavior recognition studies based on computer vision techniques, and the methods of behavior feature extraction relied on human observation and design. In this paper, the deep learning method was adopted, so there is no need to manually design feature extraction methods, and features can be learned from the data automatically. The learned features are more suitable and effective for behavior recognition. Viazzi et al. [17] divided the manual feature extraction and subsequent action classification into two separate processes. The work based on deep learning in this paper is end-to-end; pig videos were inputted and then behavior categories were outputted, which achieved a seamless connection of feature extraction and classification. Kashiha et al. [18] and Nasirahmadi et al. [8] all made the pig into an ellipse to perform image analysis and calculating, which depended on high-precision image segmentation and was susceptible to light, contrast between the pig and background, and complex backgrounds. Kashiha et al. [19] and Lao et al. [20] detected behavior through the distance between a part of the pig body and the object such as the drinking nipple and feeder, which depended on the image processing and shooting conditions. The work in this paper is not affected by light, the contrast between pig and background, and complex background, and it does not need to perform image processing on video frames.

The pig images were segmented by using a deep learning-based method in the following literatures. Zheng et al. [2] and Yang et al. [22] used Faster-RCNN to recognize pig postures and feeding behaviors. Nasirahmadi et al. [16] proposed three detector methods including Faster R-CNN, SSD, and R-FCN to recognize postures of pigs. Real-time sow drinking, urination, and mounting behavior recognition has been achieved by using an optimized target detection method based on the SSD and the MobileNet [24]. Li et al. [10] proposed Mask R-CNN to segment pigs from images and then extracted the eigenvectors for mounting recognition. These methods extracted spatial features from still images without considering the temporal features of behavior. Compared with still image classification, the temporal component in video provides an additional and important clue for recognition and behavior can be more reliably identified based on temporal information. In this paper, the spatial stream networks process image frames to get the spatial information, and the temporal stream networks process optical flow to get the motion information, so two-stream convolutional networks can extract the spatio-temporal information of the video to achieve behavior recognition. According to the experimental results of this article, the accuracy of the temporal networks is higher than the accuracy of the spatial networks. From this, we can see the importance of temporal features for recognition. Sows were segmented from all frames through the FCN model to extract spatial features; then, the temporal features were designed and extracted, and the classifier was used to classify nursing behavior finally [23]. The method of this paper can extract spatial and temporal features directly through training and is end-to-end. Another advantage of this method is that it can simultaneously identify five kinds of different behaviors that can reflect the health and welfare of pigs.

## 4. Conclusions

In this study, we established a standard pig video behavior dataset that included 1000 videos of feeding, lying, walking, scratching, and mounting from five kinds of different behavioral actions of pigs. Then, we proposed two two-stream convolutional network models including inflated 3D convnet and temporal segment networks with different network architectures for pig behavior recognition, which can get more spatio-temporal feature information of the videos. A total of 773 videos of the dataset were used to train these models, and 227 videos of the dataset were used to test these models. According to the experimental results, the average recognition accuracy of the TSN model (ResNet101) can reach 98.99%, and the average recognition time of each video is 0.3163 s. This shows that the model can extract the behavior spatio-temporal characteristics of pigs and perform classification recognition more efficiently. Pig videos are inputted and then behavior categories are outputted, realizing end-to-end. The behavior recognition method does not depend on specific pigpen distribution and has good robustness for different pig ages, pig body sizes, and lighting conditions. In the future, we will collect videos containing a larger number of pigs to study how to recognize videos containing multiple pigs, and this article lays the foundation for this future work.

## Figures and Tables

**Table 1:** The specific parameters of pig video behavior dataset. AVI: Audio Video Interleaved.

Item	Parameter
Behavior class	5
Behavior name	feeding, lying, walking, scratching, and mounting
Number	1000
Video average duration	6.276 s
Total duration	104.6 min
Resolution	320 × 240
Frame rate	25 fps
Storage format	AVI
Naming pattern	action name + number

**Table 2:** The specific network structure of Residual Network (ResNet).

Layer Name	Output Size	18-Layer	50-Layer	101-Layer
Conv1	112 × 112	7 × 7, 64, stride 2		
Conv2_x	56 × 56	3 × 3 max pool, stride 2		
		$\left[\begin{array}{c} 3\times 3,\;64\\ 3\times 3,\;64 \end{array}\right]\times 2$	$\left[\begin{array}{c} 1\times 1,\;64\\ 3\times 3,\;64\\ 1\times 1,\;256 \end{array}\right]\times 3$	$\left[\begin{array}{c} 1\times 1,\;64\\ 3\times 3,\;64\\ 1\times 1,\;256 \end{array}\right]\times 3$
Conv3_x	28 × 28	$\left[\begin{array}{c} 3\times 3,\;128\\ 3\times 3,\;128 \end{array}\right]\times 2$	$\left[\begin{array}{c} 1\times 1,\;128\\ 3\times 3,\;128\\ 1\times 1,\;512 \end{array}\right]\times 4$	$\left[\begin{array}{c} 1\times 1,\;128\\ 3\times 3,\;128\\ 1\times 1,\;512 \end{array}\right]\times 4$
Conv4_x	14 × 14	$\left[\begin{array}{c} 3\times 3,\;256\\ 3\times 3,\;256 \end{array}\right]\times 2$	$\left[\begin{array}{c} 1\times 1,\;256\\ 3\times 3,\;256\\ 1\times 1,\;1024 \end{array}\right]\times 6$	$\left[\begin{array}{c} 1\times 1,\;256\\ 3\times 3,\;256\\ 1\times 1,\;1024 \end{array}\right]\times 23$
Conv5_x	7 × 7	$\left[\begin{array}{c} 3\times 3,\;512\\ 3\times 3,\;512 \end{array}\right]\times 2$	$\left[\begin{array}{c} 1\times 1,\;512\\ 3\times 3,\;512\\ 1\times 1,\;2048 \end{array}\right]\times 3$	$\left[\begin{array}{c} 1\times 1,\;512\\ 3\times 3,\;512\\ 1\times 1,\;2048 \end{array}\right]\times 3$
	1 × 1	Average pool, 1000-d fc, softmax		

**Table 3:** Comparison of recognition accuracy under different numbers of video segment K. BN: Batch Normalization, ResNet: Residual Network.

Architectures	Pre-Training	K = 1	K = 3
BN-Inception	ImageNet	95.61%	98.42%
InceptionV3	ImageNet	96.31%	97.39%
InceptionV4	ImageNet	96.81%	97.72%
InceptionResNetV2	ImageNet	96.25%	96.32%
ResNet18	ImageNet	96.11%	98.12%
ResNet50	ImageNet	97.55%	98.55%
ResNet101	ImageNet	98.12%	98.99%

**Table 4:** Comparison of recognition accuracy with different network architectures.

Architectures	Pre-Training	Spatial ConvNets	Temporal ConvNets	Two-Stream
BN-Inception	ImageNet	91.85%	95.05%	95.61%
BN-Inception + TSN	ImageNet	94.74%	97.65%	98.42%
InceptionV3	ImageNet	94.44%	95.07%	96.31%
InceptionV3 + TSN	ImageNet	95.52%	97.22%	97.39%
InceptionV4	ImageNet	94.74%	96.02%	96.81%
InceptionV4 + TSN	ImageNet	96.02%	97.72%	97.72%
InceptionResNetV2	ImageNet	94.61%	96.02%	96.25%
InceptionResNetV2 + TSN	ImageNet	94.38%	95.38%	96.32%
ResNet18	ImageNet	94.17%	95.99%	96.11%
ResNet18 + TSN	ImageNet	94.38%	97.22%	98.12%
ResNet50	ImageNet	94.88%	97.22%	97.55%
ResNet50 + TSN	ImageNet	96.09%	98.20%	98.55%
ResNet101	ImageNet	96.46%	97.79%	98.12%
ResNet101 + TSN	ImageNet	96.32%	98.56%	98.99%

**Table 5:** The confusion matrix of the TSN model whose feature extraction network is ResNet101.

		Predicted Class				
		Feeding	Scratching	Mounting	Lying	Walking
**Actual Class**	feeding	42	−	−	−	−
	scratching	−	45	1	−	−
	mounting	−	−	49	−	−
	lying	−	−	−	55	−
	walking	−	−	1	−	34

**Table 6:** Comparison of recognition accuracy with different network parameter initialization methods. I3D: inflated 3D convnet.

Methods	Pre-Training	Spatial ConvNets	Temporal ConvNets	Two-Stream
**I3D**	−	93.30%	94.68%	95.45%
**I3D**	ImageNet + Kinetics	97.89%	98.28%	98.99%

**Table 7:** The confusion matrix of the I3D model with pre-training.

		Predicted Class				
		Feeding	Scratching	Mounting	Lying	Walking
**Actual class**	feeding	42	−	−	−	−
	scratching	−	45	−	−	1
	mounting	−	−	49	−	
	lying	−	−	−	55	−
	walking	1	−		−	34
